# Multistakeholder Perspectives on Maternal Text Messaging Intervention in Uganda: Qualitative Study

**DOI:** 10.2196/mhealth.9565

**Published:** 2018-05-10

**Authors:** Onaedo Ilozumba, Marjolein Dieleman, Sara Van Belle, Moses Mukuru, Azucena Bardají, Jacqueline EW Broerse

**Affiliations:** ^1^ Health Systems Unit Department of Public Health Institute of Tropical Medicine Antwerp Belgium; ^2^ Barcelona Institute for Global Health University of Barcelona Barcelona Spain; ^3^ Athena Institute Faculty of Science Vrije Universiteit Amsterdam Amsterdam Netherlands; ^4^ Uganda National Health Consumers’ Organization Kampala Uganda

**Keywords:** maternal health, telemedicine, community health workers, Uganda, evaluation studies

## Abstract

**Background:**

Despite continued interest in the use of mobile health for improving maternal health outcomes, there have been limited attempts to identify relevant program theories.

**Objectives:**

This study had two aims: first, to explicate the assumptions of program designers, which we call the program theory and second, to contrast this program theory with empirical data to gain a better understanding of mechanisms, facilitators, and barriers related to the program outcomes.

**Methods:**

To achieve the aforementioned objectives, we conducted a retrospective qualitative study of a text messaging (short message service) platform geared at improving individual maternal health outcomes in Uganda. Through interviews with program designers (n=3), we elicited 3 main designers’ assumptions and explored these against data from qualitative interviews with primary beneficiaries (n=26; 15 women and 11 men) and health service providers (n=6), as well as 6 focus group discussions with village health team members (n=50) who were all involved in the program.

**Results:**

Our study results highlighted that while the program designers’ assumptions were appropriate, additional mechanisms and contextual factors, such as the importance of incentives for village health team members, mobile phone ownership, and health system factors should have been considered.

**Conclusions:**

Our results indicate that text messages could be an effective part of a more comprehensive maternal health program when context and system barriers are identified and addressed in the program theories.

## Introduction

### Background

Maternal mortality is decreasing in many countries, yet over 300,000 women still die annually, particularly in low- and middle-income countries (LMICs) [1]. The utilization of mobile health (mHealth) has been proposed as a potential solution to improve maternal health outcomes in LMICs [2-4]. There is evidence in a growing body of research that despite indications of positive outcomes, the continued focus of mHealth research on activities and inputs and less on the mechanisms behind program success and failure have contributed to gaps in evidence on the effectiveness on mHealth interventions [5-8]. These authors have suggested that a greater attention to program theories could contribute to a deeper understanding of mHealth interventions.

Program theories, also referred to as theories of change, have various definitions but usually involve relating program input and activities to expected outcomes. For the purposes of this study, we approach the idea of a program theory from the perspective of “realist evaluation.” Realist evaluation emphasizes not only inputs and outcomes but attempts to answer the question of what works for whom (outcomes), how (mechanisms), and under what conditions (context) [9,10]. We did not undertake a full realist evaluation in this study; however, we utilized the principles of this form of theory-driven evaluation in understanding of the underpinnings of mHealth interventions. In our research, we aimed first to explain the assumptions of program designers, which we call the program theory; and second to contrast this program theory with empirical data to gain a better understanding of mechanisms, facilitators, and barriers related to the program outcomes.

### Intervention Description

The Ugandan National Health Commission (UNHCO), a nongovernmental organization in Kampala, designed and implemented a multisite intervention centered on the use of text messaging (short message service, SMS) for maternal health in 3 districts of Uganda. The main objective of the intervention was to improve demand and utilization of maternal sexual and reproductive health services of mothers through increased communication about recommended maternal health services and maternal health rights and responsibilities. The project took place between 2011 and 2014 in 3 districts in Uganda and was primarily targeted at women between the ages of 15 and 49 years, but other community members, including men, were also recruited onto the platform. Village health team (VHT)members received training from UNHCO and subsequently registered primary beneficiaries on an SMS platform during home visits. Primary beneficiaries then received tailored text and audio messages regarding upcoming antenatal care (ANC) visits and recommended reproductive health practices. The SMS messages were developed by UNHCO staff in consultation with maternal health professionals and Ministry of Health officials who were familiar with and worked within the local context. The messages were developed in Luganda and aimed to address evidence-based recommended health practices for improved maternal health outcome such as the need for ANC attendance and delivery and health facilities. The intervention developers also gave consideration to the rights-based approach and created messages addressing the rights and responsibilities of patients. They also included a feedback mechanism within the SMS platform, which allowed users to send feedback via free SMS messages. Table 1 contains additional information on the intervention design and implementation.

In 2015, UNHCO conducted a postintervention study and randomly sampled 1048 respondents from the intervention districts; 482 respondents were registered and 566 were not registered on the UNHCO SMS platform. Study results indicated that the SMS platform, radio programs, health workers, and VHTs had a significant joint influence on uptake of maternal health services. However, the SMS had the highest effect (odds ratio 19.086, 95% CI 10.683-34.099). Furthermore, women whose husbands received a text message within the last year were more likely to seek maternal health services compared with those whose husbands had not received messages. Despite these positive reports, there remained gaps between program developers’ expectations and the program outcomes. For example, although UNHCO received reports from health facilities that first ANC visits and delivery at health facilities had increased, the numbers remained below program designers’ expectations. In addition, only 54.4% (117/215) of the registered users surveyed indicated that they had heard about maternal health rights.

Program developers acknowledged that with consideration to these mixed results, there was a need to understand mechanisms, barriers, and facilitators to program outcomes. This study had 2 aims, first, to explicate the assumptions of program designers, which we call the program theory and second, to contrast this program theory with empirical data to gain a better understanding of mechanisms, facilitators, and barriers related to the program outcomes.

**Table 1:** Intervention description.

Input		Activities	Description
Funding		Catholic Organization for Relief Development Aid	A three and a half-year grant
**Setting**			
	Districts	In all, 3 districts selected	One rural and one semiurban district selected per district; In total, 6 subdistricts
**Intervention components**			
	Short message service (SMS) messages	Information was sent via weekly text messages on the following: antenatal care, postnatal care, immunization, nutrition, danger signs during pregnancy, delivery services, family planning, prevention of mother to child HIV, health education on self-care and child care, nutrition and breastfeeding and maternal rights and responsibilities	Women were the intended targets of the SMS messages. However, monitoring reports indicated that in some households, men owned the phones. Men were then also enrolled on the SMS platform. Messages were sent only via written text messages at first but midway through the program was modified to include voice messages also. Messages were sent in biweekly in Luganda, most commonly spoken local language in the regions. Feedback messages could be sent in response. These could be about experiences at the health center, additional questions, or issues with the SMS intervention itself. In all, 100 feedback messages were received through the SMS platform. As part of the intervention, on a quarterly basis, RH experts would develop answers to questions received through the platform, and these would be sent back as general awareness messages.
	Radio programming	Information provided based on feedback questions received through the SMS intervention	Radio programs were aired biweekly on different stations depending on the district. They addressed questions that had been sent to the SMS platform and provided additional maternal health information.
**Participants**			
	1. Village health teams (VHTs)	Prepare work plans for reaching out to the primary beneficiaries; deliver services on a reach out basis; screen, advise, and refer as required; follow-up and assess response of the primary beneficiaries to the maternal, sexual and reproductive health services	One VHT consisting of 10 individuals was invited per sub-district. In total, 60 VHT members were invited and agreed to be part of the programs. In all, two trainings per year were carried out as planned per district; one in maternal health technical content and another on the use of information communication technology tools. All VHTs received a bicycle, monthly allowance of 20,000 Uganda Shillings, and t-shirts.
	2. Primary beneficiaries	Demand and seek maternal, sexual and reproductive health services; demand for downward accountability; provide feedback on quality of service delivery	Primary beneficiaries could be men and women of reproductive age. The program reached 2341 men and women.
	3. Service providers	Provide maternal, sexual and reproductive health services; provide feedback on utilization, outcomes, and impact; provide facility data on outcomes and impact	Health workers at all health facilities within the sub districts were enrolled in the program and offered training. In total, 18 health providers were trained. However, high turnovers within health facilities disrupted the ability to measure.

## Methods

### Study Design

A qualitative retrospective study was undertaken between May and August 2016. Data collection occurred in 2 phases. The first phase involved concurrent discussions with the program developers, as well as a review of all relevant program documents. In the second phase, semistructured interviews explored perceptions of primary beneficiaries (women and men) and health service providers. Focus group discussions (FGDs) were used to investigate the perceptions of VHTs about the intervention.

### Program Theory

**Figure 1:**
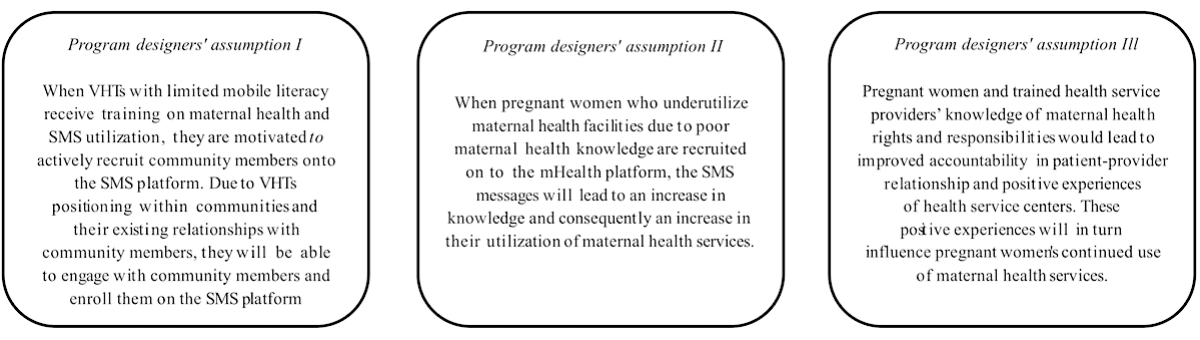
Program theory. SMS: short message service; VHTs: village health teams.

Through interviews with intervention developers (n=3) and review of the literature, we summarized the assumptions about preexisting conditions within the intervention communities and how they relate to the intervention and the expected outcomes into 3 broad statements. These statements constitute the program theory and serve as the conceptual framework for this study and are presented in Figure 1.

The intervention included a component to train VHTs. Subsequently, the VHTs were expected to recruit community members to register on an SMS platform. Linked to this was the assumption of program designers that the VHTs were motivated and that their relationships with the communities was good. If this was met, then the second assumption was that pregnant women would benefit from the text messages if their maternal health knowledge was poor and they currently underutilized health facilities. Receipt of these messages would lead to an increase in maternal health knowledge and translate to increased utilization of health facilities of this specific group of pregnant women. The third assumption was that the increased maternal health knowledge of SMS recipients and trained health service providers, particularly with respect to maternal health rights and responsibilities, would lead to positive experiences at health service centers. These positive experiences at health service centers would be crucial in sustaining improved maternal health-seeking behaviors.

### Study Population and Sampling

The study recruited participants from all 6 subcounties and 3 participant groups: primary beneficiaries (men and women), VHTs, and service providers. Criterion sampling was utilized based on the predetermined criteria listed in Textbox 1 [11].

**Inclusion criteria for study participants.**

Women

- Reproductive age (18-45 years)
- Pregnant within the intervention time frame
- Living within the intervention subcounties
- Registered on the mHealth platform

Men

- Reproductive age (18-45 years)
- Partner pregnant between 2014 and 2015
- Living within the intervention subcounties
- Registered on the mHealth platform

Village health teams

- Trained within the program
- Active within the intervention subcounties

Service providers

- Health professionals, including nurses, midwives, and doctors at the health centers in intervention subcounties who had received Ugandan National Health Commission training

#### Primary Beneficiaries (n=26)

In total, 15 women were recruited from local health centers offering maternal and pediatric care within the selected subcounties. Research assistants approached women awaiting health services at the centers, with approval from health service providers, and invited them to participate in an interview. In addition, 11 men were recruited from the community by UNHCO local supervisor and VHT program members and invited for an interview. Primary beneficiaries were most frequently farmers, with all participants having some level of formal education.

#### Village Health Team Members (n=50)

All 60 VHT members, with 20 VHT members from each district, who received the training, were invited to participate in an FGD, of whom 50 responded. District supervisors explained that the nonresponding VHT members were invited but were generally considered to be inactive, both with respect to the intervention as well as their general duties. In total, 6 FGDs were conducted with 50 VHT members in the 6 subcounties (5 of the 6 FGDs had 6-8 participants, while 1 FGD had 10 participants). All VHT members interviewed had a primary occupation, which ranged from paid positions within the health system to farming.

#### Health Service Providers (n=6)

The 18 trained health service providers who were involved in the program training and supervision of VHTs were eligible to participate. However, due to frequent staff turnover, we were only able to contact 6 health service providers who were involved in the intervention; all agreed to participate in an interview.

#### Data Collection

Interviews with primary beneficiaries were conducted with the aid of vignettes [12], while semi structured guides were used for the FGDs with VHTs and service providers. Guides were pilot-tested in another district not involved in the project, and corrections were made. General themes for all participant groups included maternal health-seeking behavior, relationships between VHTs and communities, and experiences with the SMS intervention. Additionally, VHTs and health service providers were asked about their experiences with training. FGDs lasted between 90 and 120 min, and individual interviews ranged between 30 and 60 min.

#### Data Management and Analysis

All interviews were conducted, recorded, and transcribed by 2 local research assistants. Verbatim translation and transcription were conducted from Luganda (local language) to English and analyzed in MAXQDA version 12 (VERBI GmbH, Germany).

Data analysis was executed by means of content analysis for which a codebook was developed based on the conceptual framework and research questions. On the basis of the assumptions (conceptual framework) and research questions, an initial codebook was designed [13]. After data collection, 2 researchers utilized this framework to code one purposively selected data rich transcript from each participant group. The codebook was then modified based on conversations between both researchers. During the coding process, codes were reviewed and when necessary, codes were revised and new codes that emerged from the data were added. In the code scheme, each code was categorized under a corresponding theme, and quotes from the participants were collected throughout the coding process. Themes were identified in a deductive manner, using the conceptual framework as a guide. All coding and analysis were performed in MAXQDA version 12 (release 12.1.4).

### Ethical Considerations

The study was granted ethical approval by the Vrije Universiteit Amsterdam, the Netherlands, and Makerere University, Uganda. Depending on the literacy level of the participant, fingerprint or written consent was collected from participants. The interviewer/translator read the consent form in the appropriate language to the participant. Assurance was provided to participants that data collected would be used for research purposes only. No identifiable personal data or health data were collected from participants. All participants were offered refreshments for their participation in the interviews. This was deemed appropriate and not significant enough to be coercive within the local context.

## Results

### Program Designers’ Assumption I

For VHTs to perform their duties according to the expectations of program designers, training is essential, particularly in a context where VHTs themselves may be unable to navigate mobile phone functionalities such as text messaging. However, in addition to the contents of the training program, factors such as the duration and frequency of training, as well as the provision of incentives and the relationship between VHTs and their communities contributed to the expected outcome of Assumption I.

#### Perceptions of Training

Overall, most of the VHT members interviewed had positive accounts of the training they received. VHT members explained that before the training they had limited knowledge about reproductive health issues, including family planning and ANC, and maternal or sexual and reproductive health rights and responsibilities. All of the VHT members reported that they were unable to send text messages before the training:

When UNHCO was training, they used chats, they asked us questions to find out how much we know and before they introduced a topic, they always tested our knowledge about it. Secondly, they taught us how to send messages from our phones and we understood it well and we also in turn went back into the community to teach the same information on how to utilize their phones in health messages.

FGD 4, Participant 5

The notable complaint was with the timing, frequency, and consistency of trainings, which varied between groups. Four of the VHT members particularly found these inconsistencies to affect their learning.

Due to high staff turnover, not all health workers in the intervention districts benefited from the training. Only one health service provider in each of the districts indicated that they received training by UNHCO. The other 3 health workers had been involved in meetings and were aware of the SMS intervention but had not received any training. The 3 health service providers who had received training had positive perceptions of the trainings they had participated in.

#### The Role of Incentives

In all FGDs, VHT members discussed the importance of incentives. Incentives were discussed as the unmet need for incentives (as well as the fact that incentives had been promised but were not awarded). In general, VHT members linked their ability to utilize what they learned in the training to the enabling conditions provided through the incentives offered to them by the nongovernmental organization. These incentives were both financial and nonfinancial and included financial compensation for transportation or food, bicycles, rain boots, and t-shirts. VHT members viewed the provision of incentives as inadequate:

We also, as the advocates are inadequately facilitated. We don’t get fed in meetings and we receive only a small allowance to facilitate us back to our residencies. So UNHCO has to change its approach first in the handling of its advocates. We serve a radius of 15 kilometers of each of the two villages, so it has to polish on the transportation within those areas of operation.

FGD4, Participant 6

VHT members were also critical about what they perceived as unfairness in the distribution of the incentives that were provided. For example, only a handful of people received bicycles. They viewed this as favoring one VHT member’s work over the other. However, UNHCO records indicated that all VHT members received a bicycle, a monthly allowance of 20,000 Uganda Shillings (equivalent to US $5), and t-shirts. We could not identify the underlying reasons for these differences in VHT management.

Additionally, 2 groups of VHT members also discussed the need to incentivize participation on the SMS platform. The primary beneficiaries, mothers and fathers in the communities, on the contrary, did not express any need to be incentivized to participate in the SMS intervention. The two most frequently cited reasons of primary beneficiaries for registering on the mobile platform were that the messages were free and would improve quality of maternal and neonatal health outcomes:

Reason number one is that when they were registering us, they told us these messages are for free with no cost attached. Secondly these were health messages and I thought when I inform others the number of death rates in our community would reduce.

Male participant 4

#### Relationships Between Village Health Teams and Communities

VHT members reported a positive relationship with communities. Some VHT members shared previous negative experiences. These poor relationships were related to communities’ initial resistance of VHT instructions, a belief that the VHTs received financial compensation for the work, mockery because they were not health professionals, or a perception that the VHTs were nosy. However, these views reportedly changed with evidence of positive outcomes arising from VHTs’ activities. Many VHT members felt increasingly valued by the communities:

For example, when I had just joined VHT I had a neighbor who used to mock me saying “what can that one do” but one day one of the grandchildren she was staying with fell sick…I tested the child and gave her medicine and when the grand child was better the following day, she started spreading the news that VHTs are real doctors…The relationship is generally good, apart from a few individuals.

FGD 6, participant 4

For the trust relationship between VHTs and the communities, all but 3 of the primary beneficiaries expressed trust in the VHTs. Among those who indicated low levels of trust the major reason was a belief that VHTs did not maintain confidentiality. Those who expressed trust in VHTs cited reasons ranging from the selection process for VHTs, preexisting relationships between VHTs and women, and women’s perception of the usefulness of the maternal health information provided by VHTs:

These VHTs were selected by the people of this community. They are people who are committed to doing volunteer work… Because women elected them, it makes it easier to trust.

Woman, participant 6

### Program Designers’ Assumption II

While maternal health knowledge is an important factor in maternal health-seeking behavior, additional relevant factors influenced community members’ ability to engage with the intervention such as literacy level, mobile phone ownership, and male involvement in reproductive health decision making.

#### Literacy

The concept of an SMS platform was well received by the community and there were no complaints about network or power outages in relation to using mobile phones or receiving text messages. However, high illiteracy levels in the implementation district meant that SMS messages had a limited impact. The introduction of voice messages was a welcomed improvement to the program:

The voice messages are very good because at least all people apart from the deaf have ears and therefore can listen directly to what is being said and understand it well but written messages are limited to the educated.

Woman, participant 22

An unintended effect of primary beneficiaries’ limited literacy was that women would sometimes consult VHT members when they received messages. This was raised by VHT members themselves, as well as by most of the primary beneficiaries who suggested that women who could not read the SMS could ask their VHTs for help.

#### Mobile Phone Ownership

For an SMS intervention that does not distribute free mobile phones to have the intended impact, it was essential that the intended message recipients already possessed mobile phones. However, about half of the study respondents indicated that they or their spouses did not possess personal mobile phones. Of the participants who indicated that they or their wives owned a personal mobile phone, low mobile literacy among women was a frequently cited problem. Low mobile phone ownership and literacy among women meant that the messages had to be received on their husband’s, neighbor’s, or a friend’s mobile phone. When men received the messages, there was an unintended positive outcome of men getting more involved in maternal health care:

Since in most homes it is the men who have phones, these messages used to come directly to them and in the long run it has also improved male involvement in maternal health issues.

FGD6, participant 3

#### Male Involvement in Reproductive Health Decision Making

Participants defined male involvement as men having knowledge about required maternal health behavior and taking an active role in their pregnant spouse’s health seeking. This included attending ANC visits, getting tested for HIV and other sexually transmitted infections, as well as supporting their partners in their decision to seek care.

Although men could be and were registered on the SMS platform as primary beneficiaries, they were not the intended target group. However, study participants frequently discussed the importance of male involvement in reproductive health-seeking behaviors, and husbands were often mentioned in the interviews as the decision makers. Husbands’ objections to HIV testing, family planning, and facility delivery were frequently cited as potential barriers to women’s ability to seek reproductive services at health facilities:

I have an example, a woman had not attended any antenatal services by the time she was seven months pregnant. When I asked her why she was not going to the hospital, she said her husband had not given her permission to go to the hospital and (there was) no transportation money

FGD 3, participant 5

Most of the participants—primary beneficiaries, VHT members, and health service providers—acknowledged that it was important for a woman to have an input in her health care decision making and that maternal health decisions should be made jointly. The majority of the men interviewed indicated that they had now started taking joint health decisions with their wives and were more involved in her maternal health. There was a general consensus that overall the situation had improved since men started receiving the SMS messages.

### Program Designers’ Assumption III

Results showed indications of improved accountability in client-provider relationship and increased positive experiences of primary beneficiaries at the health centers. However, our results highlighted significant individual and health barriers that influenced participants’ experiences at health centers.

#### Individual-Level Factors

In this study, individual factors identified were knowledge of sexual and reproductive rights and responsibilities, and the relationships between primary beneficiaries and health service providers.

#### Knowledge of Sexual and Reproductive Rights and Responsibilities

Client’s knowledge of their rights and responsibilities included the right to know the medical professionals’ name, the results from the medical checkup, and to get proper prescription of medicine. Responsibilities included, following the health providers’ instructions and taking medication as directed. Program developers expected that this knowledge of rights would influence relationships between service providers and their clients. All VHT members and primary beneficiaries interviewed could name at least one health right and responsibility, and they attributed this knowledge to their training or receipt of SMS messages. VHT members and health service providers shared that when women knew their health rights and responsibilities, it reduced their workload and improved their ability to provide care. VHT members also emphasized that this knowledge had helped improve the relationship between health service providers and clients:

Having knowledge about patients’ health rights has helped us to bridge the gap between the health professions and the community members because everyone is aware of what they must do…

FGD6, participant 8

Primary beneficiaries agreed that the text messages had improved their knowledge about rights and responsibilities and empowered them to demand answers and appropriate treatment from the health professionals. Although VHTs and health service providers shared multiple anecdotal stories of their community members demanding treatment and actions from health service providers based on their knowledge, only one participant shared an example of actively demanding her rights when a health service provider attempted to treat her poorly.

#### Relationships Between Primary Beneficiaries and Health Service Providers

Despite the increase in the knowledge on health rights and responsibilities, in all interviews with VHTs, there were reports of poor treatment of clients by health service providers. However, in all but one of the intervention districts, the situation was reported to have improved considerably during the intervention period. Reasons for this improvement varied, but overall the consensus was that training of health professionals and informing primary beneficiaries of their health rights and responsibilities through the SMS platform contributed to better treatment of clients:

There is a large difference because those who are registered [on the SMS platform] are no longer afraid to come to the hospitals but some of the people who are not registered have a negative attitude towards hospitals.

FGD 4, participant 2

Responses from primary beneficiaries were similar to those of the VHTs. There were multiple accounts of past experiences with unpleasant or harsh health professionals. Although most participants reported improved treatment of clients, a few respondents from one district discussed the continued presence of disrespectful care from health service providers; this included speaking to pregnant women sarcastically or ignoring them.

#### At the Level of the Health System

Relevant factors that were classified at the level of the health system were related to the integration of levels of care and existing barriers to accessing care

#### Contributed to Better Integration of Levels of Care

The intervention contributed to improved referral. Primary beneficiaries in the intervention districts received their maternal health care from 2 levels of service providers: VHTs and health service providers (nurses, midwives, medical doctors). For participants to have positive experiences of maternal health services, the integration of these 2 levels was important. Overall primary beneficiaries, VHTs, and health service providers judged the working relationship between VHTs and health service providers positively. VHT members shared that health workers treated them as colleagues, respected their input, and worked well with them. Program beneficiaries echoed the positive collaboration between VHTs and health service providers to achieve the mutual objective of improved maternal health service delivery. The beneficiaries’ experiences were related to observing the daily interactions between VHTs and health service providers; the perception that VHTs brought health education to communities on behalf of health service providers and finally, the understanding that VHTs reduced the work load of health service providers. For example, when VHTs thought that a woman needed professional medical care, they issued referral forms. Women in possession of these forms experienced that health service providers promptly addressed their cases. More effective referral systems can be considered indicative of improved health service delivery:

They treat her well and work with her very quickly once they have seen the referral from the VHT. The people who come with these referral forms don’t even wait in the line. They are worked upon as soon as they reach the hospital.

Woman, participant 10

#### Barriers to Accessing Care

Participants also highlighted other system-level factors that influenced their overall health service experience. These included difficulties in reaching health facilities due to transportation issues and referrals to far-away district hospitals. Although UNHCO provided “mama kits” that contained required items such as bandages and clothing, this was not standard practice for every patient, and many respondents reported insufficient funds to purchase the required items (bandages and clothing) as a barrier to access care. Women shared their view that an inability to purchase these items could result in harsh words or treatment from health workers and could discourage women from seeking care. In addition, problems at the health facility, such as drug supply stock outs, lack of ambulances, staff shortage, and poorly maintained or no equipment, negatively affected experiences of maternal health seeking. The SMS intervention was not geared at fixing these health systems–related issues, but they arose consistently in interviews with all study participants as significant barriers in accessing care and their experiences of care received at health facilities.

## Discussion

### Principal Findings

**Figure 2:**
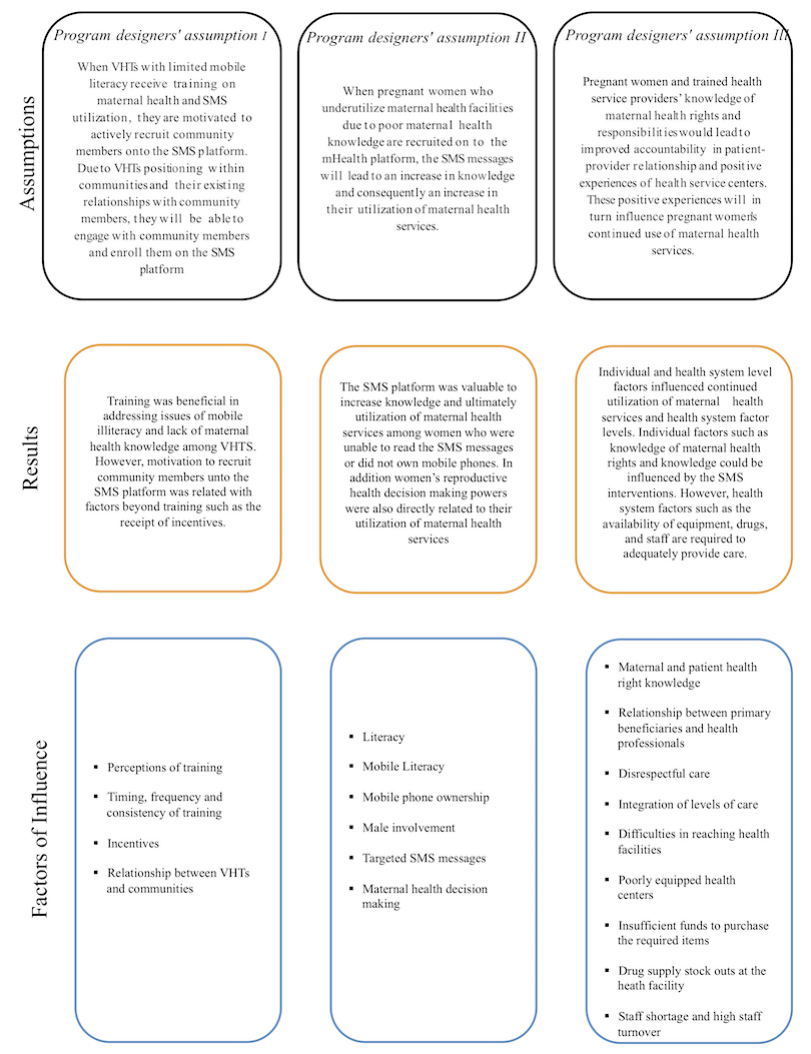
Revised theory of change. SMS: short message service; VHTs: village health teams.

The overall goal of the maternal SMS platform was to create an intervention in line with national health policies to improve demand and utilization of maternal health services at the community level. This paper sought to provide an additional understanding of the program outcomes by analyzing the fit between 3 implicit assumptions of a maternal SMS intervention and perceptions of program participants, including primary beneficiaries, VHTs, and health service providers. In our study, program developers’ assumptions were partially supported; however, relevant mechanisms, facilitators, and barriers related to the program outcomes were identified. These are presented in Figure 2 and discussed further within this section.

Program developers assumed that training of VHTs would result in motivated VHTs who engaged with the mHealth platform. Our study found that while training was important to ensure that VHTs had the necessary knowledge, other incentives were also very relevant. VHT members discussed the importance of receiving the promised incentives and the desire for additional incentives. Previous research on the importance of incentives for community health workers (CHWs), which VHTs are classified as, have shown that when CHW perceive their incentives as not commensurate to their efforts or when the incentives are not delivered consistently, motivation is negatively affected [14,15]. Despite the sustained conversation around incentives, the impact of remuneration in mHealth interventions generally focuses on the need of funds for airtime or data purchases on mobile phones [16,17]. It must be noted that UNHCO’s incentive delivery records and the report of VHTs were misaligned, but this could not be further explored in this study.

In the program designers’ first assumption, VHTs by virtue of the training received and their position in the communities would be able to recruit primary beneficiaries onto the SMS platform. Our results highlight that the relationship between VHTs and community members is influenced by various factors. This included previously researched factors, such as the selection process of VHTs, community members’ trust in VHTs, and community members’ perception of the suitability of selected VHTs to provide services [18]. Consistent with findings by Musinguzi (2017), participants in our study, including VHTs, had past negative experiences with health professionals [15]. However, these experiences decreased during intervention implementation and contributed to a joint approach to providing health services; VHT members expressed that they felt the health service providers appreciated their work. Despite the intricacies of the relationship between VHTs and community members, our results did not indicate that there were any existing problems, which influenced the overall enrollment onto the SMS platform.

The second assumption—women’s health-seeking behavior would improve based on information received from the SMS platform—was inconclusive due to contextual factors beyond maternal health knowledge that influenced women’s health-seeking behavior. The 3 critical barriers observed were low literacy, the gender differential in mobile phone ownership, and males as decision makers in matters related to reproductive health. The effects of low literacy levels, including mobile literacy, have been highlighted as a limitation of program outcomes for health workers and patients in Uganda and similar countries [19,20,4]. UNHCO recognized low literacy level as a barrier and modified the platform to include voice messages, which primary beneficiaries and VHTs reported to be beneficial. However, the issues of male mobile phone ownership could not be addressed directly in this intervention. The intervention was modified to allow husbands to receive the text messages, but as they were not the intended targets, the messages were not tailored for them. However, the inclusion of men on the SMS platform had the unintended positive effect of improving male involvement in maternal health decision making. Given the higher levels of mobile phone ownership by men and the common role of men as decision makers in the intervention districts, these could be significant in understanding why ANC attendance and facility delivery increased, although the program designers’ expectations were not met.

Finally, the third assumption explored the idea that an increase in knowledge of maternal health rights and responsibilities would lead to improved accountability in client-provider relationship and consequently positive experiences of health service centers and continued utilization of these services. However, the interviews revealed that while this knowledge was helpful and did lead to some improvements in experiences, other health system factors still negatively influenced health service experiences and continued utilization of services. These factors included accounts of mistreatment of pregnant women, payment of bribes, insults to women, and harsh treatment by health professionals—factors which were also found in previous research [21,22]. This lack of change could, in part, be ascribed to the high turnover of health service providers, which meant that a limited number (35%) of the staff in the intervention districts had actually received training on maternal rights and responsibilities. There were also issues around the availability of equipment and drugs that meant that services, which were meant to be fee exempted, still had costs. These findings indicate that while the cooperation between health service providers, VHTS, and primary beneficiaries improved, barriers to seeking care still exist and these could have impacted the intervention outcomes.

### Limitations

The main limitation of this study is that while this intervention assessed perceptions of VHTs and primary beneficiaries of the SMS platform, UNHCO had other ongoing programs in the same sites. Some of these were specifically related to maternal health accountability, such as the installation of suggestion boxes at health facilities and the training of maternal and perinatal death review committees. Furthermore, the health facilities had to close down due to theft and reopened after renovations. The multiplicity of interventions as well as the time elapsed (retrospective design) since the end of the intervention might have affected the recall of participants. To address this, participants were consistently probed and asked follow-up questions to ensure that they were referring to the SMS intervention. Another possible limitation arises from the decision to utilize health centers as the recruitment location of female primary beneficiaries. This might mean that our respondents are a select subgroup who actively utilize health services. However, recent statistics report high attendance of at least one ANC and high uptake of infant vaccination [23]. Thus, clients at health center during routine clinic hours were likely to represent a spectrum of health-seeking behaviors such as those found in the general population. In addition, within our sample, we conducted interviews until no new information emerged, ensuring that data saturation was reached. As with all research that relies on self-reported data, there was a risk that participants gave socially desirable answers. We attempted to minimize this risk by reassuring participants that all the information they provided would be kept confidential. We also ensured that no staff member of the UNHCO team or health center was within the vicinity when the interviews took place. Finally, we were unable to test the assumptions raised in this study as we focused more on understanding how the assumptions played out during intervention implementation. This could be an interesting starting point for future research. For example, future research could involve systematically assessing the relationship between VHTs and their community members and the effect of this on intervention outcomes.

### Conclusions

In reflecting on the role of SMS messaging as a tool to achieving maternal health outcomes, some key messages emerged. The utilization of a program theory allows for the unpacking and testing of intervention designers’ assumptions. Our results highlight the importance of using program theory to highlight the need to address the broader contextual factors that could limit the achievement of their intended mHealth outcomes. In contexts when there are health system barriers, such as informal payments, frequent staff turnover, drug stock outs, and the lack of integration of service platforms, the study shows that program designers could adapt program outcome expectations or modify the intervention to address these system factors. We conclude that SMS interventions such as this could be most effective when incorporated in comprehensive multilevel programs, which address health system barriers and constraints.

## Abbreviations

ANC: antenatal Care
CHWs: community health workers
FGD: focus group discussion
LMIC: low- and middle-income countries
mHealth: mobile health
SMS: short message service
UNHCO: Ugandan National Health Commission
VHTs: village health teams
